# Ceftriaxone‐associated pseudolithiasis

**DOI:** 10.1002/jgf2.435

**Published:** 2021-03-24

**Authors:** Masaru Kurihara, Yasuharu Tokuda

**Affiliations:** ^1^ Department of Hospital Medicine Urasoe General Hospital Okinawa Japan

**Keywords:** ceftriaxone, pseudolithiasis

## Abstract

Ceftriaxone‐associated pseudolithiasis.

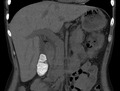

A 52‐year‐old woman developed acute onset abdominal pain. She was previously prescribed ceftriaxone (CTRX) 2.0 g IV q12H for an epidural abscess and had been receiving this for 41 days. She had no history of renal failure. On examination, her temperature was 36.8°C. She presented with tenderness in the right lower quadrant of the abdomen, although the peritoneal sign was absent. Laboratory blood test results were within normal ranges, aside from an elevated C‐reactive protein level of 11.0 mg/dL. Abdominal contrast‐enhanced computed tomography (CT) revealed a high‐density lesion in the gallbladder (Figure 1) that was not observed on the CT scan on admission. A diagnosis of CTRX‐associated gallbladder pseudolithiasis was made, and the antimicrobial was changed from CTRX to cefotaxime 1.0 g IV q8H for 10 weeks. She was discharged at 81 days without recurrence of abdominal pain, and an abdominal CT scan four weeks later showed that the pseudolithiasis was gone.

**FIGURE 1:**
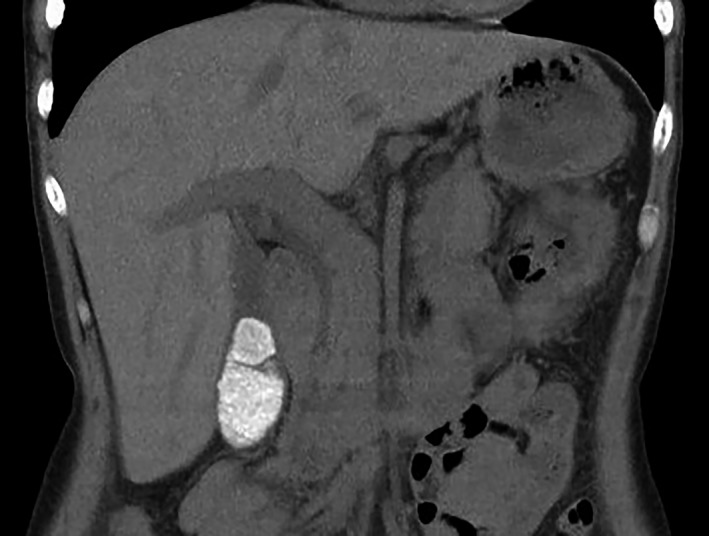
Abdominal contrast‐enhanced CT revealed a high‐density lesion in the gallbladder indicating ceftriaxone‐associated pseudolithiasis

The incidence of pseudolithiasis in adult patients is about 20% on CTRX, but only 30% of these patients present with clinical symptoms^1^. High‐dose CTRX, long‐term CTRX treatment, fasting, total parenteral nutrition, and renal failure have all been reported as risk factors for CTRX‐induced pseudolithiasis ^2^. This is rarely a clinical problem, as most cases resolve spontaneously after withdrawal of CTRX ^3^.

## CONFLICT OF INTEREST

The authors declare no conflict of interest for this report.

## INFORMED CONSENT

Informed consent was obtained from the patient for publication of this report.
